# P148: Hand hygiene communication from healthcare workers to patients: results of a pilot survey in several healthcare facilities

**DOI:** 10.1186/2047-2994-2-S1-P148

**Published:** 2013-06-20

**Authors:** D Verjat-Trannoy, A Gauthier, M-A Ertzscheid, N Jouzeau, S Monier, D Zaro-Goni, P Astagneau

**Affiliations:** 1 CCLIN PARIS-NORD, Paris, France; 2 CCLIN OUEST, Rennes, France; 3 CCLIN EST, Nancy, France; 4 CCLIN SUD-EST, Lyon, France; 5 CCLIN SUD-OUEST, Bordeaux, France

## Introduction

Hand hygiene (HH) improvement requires a joint involvement of caregivers and patients. Asking patients to remind caregivers to perform HH is not so easy to perform because of possible damage in relationship quality. Another approach giving caregivers communication initiative could improve both patient awareness and caregiver’s commitment in HH promotion.

## Objectives

In 2012, a pilot study was proposed to voluntary healthcare facilities (HCF): an evaluation was carried out in order to test the feasibility and the benefits of such an approach.

## Methods

At patient arrival, staff member was asked to implement three actions: verbal information on HH (about what is required for caregivers and patients), hand rub technique demonstration and HH flyer distribution. Two questionnaires (patient/caregiver) were provided to the participating HCF. Data collected were centralized in order to perform a descriptive analysis.

## Results

The collected results concern 8 public or private HCF and 46 clinical wards. *Patients*: 270 patients received HH information and 98% of them completed the questionnaire. This approach was considered as beneficial by 92% of them: increase of their knowledge and easier communication with caregivers. 92% of patients were willing to pay more attention to their own HH. *Caregivers*: over the 69 healthcare workers (HCW) participating, professionals or students, 91% completed the questionnaire. Most of them easily adhered to the approach (90%) and communicated to patient without any difficulty (89%). The time spent for this communication was estimated at less than 5 minutes by 37% of HCW. 78% of the HCW felt that this approach was appreciated by patients. 40% of HCW reported a change in their own HH behavior. 98% considered that this communication should be integrated in patient care and 91% thought it could be routinely performed.

## Conclusion

We note a direct benefit for patients who are rather “pleasantly surprised”. This communication also takes parts in the individual and collective engagement of caregivers about HH by making them do what they declare. We wish to propose this “easy to implement” approach to a greater number of HCF in May 2013.

## Disclosure of interest

None declared

